# Whole-exome sequencing of calcitonin-producing pancreatic neuroendocrine neoplasms indicates a unique molecular signature

**DOI:** 10.3389/fonc.2023.1160921

**Published:** 2023-09-12

**Authors:** Claudia Döring, Katharina Peer, Katrin Bankov, Carmen Bollmann, Annette Ramaswamy, Pietro Di Fazio, Peter Johannes Wild, Detlef Klaus Bartsch

**Affiliations:** ^1^ Dr. Senckenberg Institute of Pathology, University Hospital Frankfurt, Frankfurt am Main, Germany; ^2^ Department of Visceral, Thoracic and Vascular Surgery, Philipps-University Marburg, Marburg, Germany; ^3^ Institute of Pathology, Philipps-University Marburg, Marburg, Germany; ^4^ Frankfurt Institute for Advanced Studies (FIAS), Frankfurt am Main, Germany

**Keywords:** whole exome sequencing, Calcitonin-producing pancreatic neuroendocrine neoplasms, *MEN1*, *ATRX*, *PIK3CA*, *MUC4*, *MUC16*

## Abstract

**Introduction:**

Calcitonin-producing pancreatic neuroendocrine neoplasms (CT-pNENs) are an extremely rare clinical entity, with approximately 60 cases reported worldwide. While CT-pNENs can mimic the clinical and diagnostic features of medullary thyroid carcinoma, their molecular profile is poorly understood.

**Methods:**

Whole-exome sequencing (WES) was performed on tumor and corresponding serum samples of five patients with increased calcitonin serum levels and histologically validated calcitonin-positive CT-pNENs. cBioPortal analysis and DAVID gene enrichment analysis were performed to identify dysregulated candidate genes compared to control databases. Immunohistochemistry was used to detect the protein expression of *MUC4* and *MUC16* in CT-pNEN specimens.

**Results:**

Mutated genes known in the literature in pNENs like *MEN1* (35% of cases), *ATRX* (18-20% of cases) and *PIK3CA* (1.4% of cases) were identified in cases of CT-pNENs. New somatic SNVs in *ATP4A, HES4*, and *CAV3* have not been described in CT- pNENs, yet. Pathogenic germline mutations in *FGFR4* and *DPYD* were found in three of five cases. Mutations of *CALCA* (calcitonin) and the corresponding receptor *CALCAR* were found in all five tumor samples, but none of them resulted in protein sequelae or clinical relevance. All five tumor cases showed single nucleotide variations (SNVs) in *MUC4*, and four cases showed SNVs in *MUC16*, both of which were membrane-bound mucins. Immunohistochemistry showed protein expression of *MUC4* in two cases and *MUC16* in one case, and the liver metastasis of a third case was double positive for *MUC4* and *MUC16*. The homologous recombination deficiency (HRD) score of all tumors was low.

**Discussion:**

CT-pNENs have a unique molecular signature compared to other pNEN subtypes, specifically involving the *FGFR4, DPYD, MUC4, MUC16* and the *KRT* family genes. However, a major limitation of our study was the relative small number of only five cases. Therefore, our WES data should be interpreted with caution and the mutation landscape in CT-pNENs needs to be verified by a larger number of patients. Further research is needed to explain differences in pathogenesis compared with other pNENs. In particular, multi-omics data such as RNASeq, methylation and whole genome sequencing could be informative.

## 1 Introduction

Pancreatic neuroendocrine neoplasms (pNENs) are a relative rare malignancy with an annual incidence of approximately 1/100.000/year (1). pNENs can be classified as either nonfunctional (approximately 60%–70% of cases) or functional (30%–40% of cases) tumors, depending on their ability to secrete biologically active hormones. Elevated hormone levels in the serum have the potential to cause clinical symptoms, such as hyperinsulinism in case of insulinoma (1). Insulinoma (70%) and gastrinoma (20%) are the most frequent functional pNENs, while the remaining so-called rare pNENs include various hormones (5%–10%), such a vasoactive polypeptides (vipoma), glucagon (glucagonoma), serotonin, renin, and *GLP1* (glucagon-like peptide 1) (1). As of 2017, very few case reports or small case series of calcitonin-producing pNENs (CT-pNENs) have been reported, amounting to approximately 60 cases worldwide (2–18). Calcitonin (CT) is a polypeptide usually released from the C cells of the thyroid gland (8). Elevated serum CT levels are highly suggestive for the presence of a medullary thyroid carcinoma (19). Differential diagnosis of serum hypercalcitoninemia also includes alcoholism, use of medication, renal failure, bacterial infection, and hypercalcemia (20). A recent pathological study showed that approximately 10% of all pNENs, functioning and nonfunctioning, reveal a positive calcitonin immunoreactivity (9), whereas the corresponding serum CT levels were either not reported or within the normal range. The genetic alterations underlying these extremely rare CT-pNENs have not yet been explored. Therefore, in addition to conventional histopathological examination, we performed whole-exome sequencing (WES) of DNA isolated from serum (normal control probes) and tumor probes of five patients affected by CT-pNEN and compared results to available whole-exome databases. WES can reveal mutations in the coding exon regions. We hypothesized that the molecular mechanism of CT-pNENs might be related to a unique molecular signature of mutations. To validate candidate genes, cBioPortal analysis, Human Protein Atlas (HPA), and DAVID gene enrichment analysis were performed. Immunohistochemistry (IHC) was used to detect the protein expression of candidate genes in CT-pNEN samples. Our study reveals for the first time the molecular signature and features of this rare disease.

## 2 Materials and methods

Patients diagnosed with CT-pNENs were identified from the prospective pancreatic database of the Department of Visceral, Thoracic and Vascular Surgery, Philipps-University Marburg, which was established in 2008 as a prerequisite for certification as a Center of Excellence for Pancreatic Surgery by the German Society for General and Visceral Surgery (DGAV). Clinicopathologic data of the identified patients were retrospectively evaluated. The diagnosis of CT-pNEN was defined as a neoplasm associated with elevated serum CT levels and positive immunostaining for CT. The fact that the elevated CT serum levels before surgery reverted back to normal levels after surgery suggested pNEN as the source of CT secretion. All patients gave informed consent for the genetic analysis, and the study was approved by the ethics committee of the University of Marburg (No:206/10 and No:104/99). The clinical data of one patient have already been reported previously (8, 10).

### 2.1 Whole-exome sequencing

#### 2.1.1 Genomic DNA isolation from patients’ probes

The genomic DNA was isolated from tumor tissue and the corresponding serum (normal control) of five patients with a CT-pNEN.

Genomic DNA was extracted from five resected tumor tissues and corresponding serum (200 µL) snap-frozen/stored in liquid nitrogen probes by using the NucleoSpin Tissue, Mini kit (740952.50, MACHEREY-NAGEL GmbH & Co. KG, Düren, Germany). The isolated genomic DNA was further incubated for 15 min at room temperature with 1 µg/150 µL of RNAse A (EN053, Thermo Fisher Scientific, Waltham, MA, USA) and processed with NucleoSpin gDNA Clean−up, Mini kit for DNA clean up and concentration (740230.50, MACHEREY-NAGEL GmbH & Co. KG). The amount and purity (260/280 nm) of DNA were measured by NanoDrop. The integrity of the isolated DNA was performed by gel electrophoresis [1.0% agarose gel; 1.0% Tris-acetate-EDTA (Sigma-Aldrich, St. Louis, MO, USA) solution] at 100 V for 40 min. One microgram (20 ng/µL) of genomic DNA of each sample was analyzed by Novogene Co., Ltd. (Cambridge, CB4 0FW, United Kingdom) for sequencing and primary analysis.

#### 2.1.2 Library preparation

In this study, 1.0 µg DNA per sample was used for library preparation. Sequencing libraries were generated using Agilent SureSelect Human All Exon kit (Agilent Technologies, CA, USA) following the manufacturer’s recommendations, and index codes were added to each sample. Captured libraries were enriched by PCR to add index tags to prepare for hybridization. Resulting products were then purified using the AMPure XP system (Beckman Coulter, Beverly, USA) and quantified using the Agilent high-sensitivity DNA assay on the Agilent Bioanalyzer 2100 system.

#### 2.1.3 Clustering and sequencing

The clustering of the index-coded samples was performed on a cBot Cluster Generation System using TruSeq PE Cluster Kit v4-cBot-HS (Illumina, San Diego, USA) according to the manufacturer’s instructions. After cluster generation, the libraries were sequenced on Illumina’s HiSeq 2000 sequencing platform.

#### 2.1.4 Sequencing data analysis

Raw sequencing data were filtered using the following procedure. Discard a read pair if either one read contains adapter contamination or more than 10% of bases are uncertain in one read or the proportion of low-quality bases is over 50% in one read. Sequencing error rate examination, GC content distribution, sequencing quality distribution, and statistics of sequencing quality (Q30 >80%) criteria are calculated for all samples. Read sequences were mapped to the human reference genome (GRCh38/hg38) using Burrows–Wheeler Aligner (BWA, v. 0.7.17) with the default parameters, and duplicates were marked and discarded using Picard (http://broadinstitute.github.io/picard, v. 2.18.9). Single-nucleotide variants (SNVs) and small insertions and deletions (INDELs) from BAM files were performed using Genome Analysis Toolkit (GATK, v. 4.0). ANNOVAR (v. 2015Dec14) was utilized to annotate all called variants.

The SNVs of the serum samples in CT-pNEN samples were removed for further analysis. The minor allele frequency (MAF) of variants was evaluated in the 1000 Genome Project (http://www.ncbi.nlm.nih.gov/variation/tools/1000genomes/) and ExAC (http://exac.broadinstitute.org/), and variants of MAF <1% and annotated by “.” (No annotation information in the database) were retained. Subsequently, the pathogenicity of variants was predicted according to SIFT (http://sift.jcvi.org), Polyphen2 (http://genetics.bwh.harvard.edu/pph2), and FATHMM (http://fathmm.biocompute.org.uk/). Variants predicted by the three tools as “D (damaging)” were retained for gene set enrichment analysis. The ClinVar database (21) archives and aggregates information about relationships among variations and human health. The ClinVar pathogenic SNVs were filtered with clinical significance for this single variant from each tumor sample.

### 2.2 Homologous recombination deficiency score

From the output of the R package Sequenza, a homologous recombination deficiency (HRD) score was calculated using the R package scarHRD (22, 23). Estimates of the global levels of loss of heterozygosity (LOH), the number of large-scale state transitions (LSTs), and the number of telomeric allelic imbalances (TAIs) were calculated separately, and the unweighted sum of these was defined as the HRD score. A score of >42 was used as the cutoff for high HRD, as defined in breast cancer (24, 25).

### 2.3 Functional gene enrichment analysis

The online biological tool DAVID 2021 Update (26) was used to analyze the molecular and functional characteristics of the SNVs. The Database for Annotation, Visualization and Integrated Discovery (DAVID) is a free online database that provides a comprehensive set of functional annotation tools for investigators to understand the biological meaning behind large lists of genes. In the present study, we use all germline variants of each sample predicted as damaging according to SIFT (http://sift.jcvi.org), Polyphen2 (http://genetics.bwh.harvard.edu/pph2), and FATHMM (http://fathmm.biocompute.org.uk/) for functional gene enrichment analysis.

### 2.4 Immunohistochemistry

Snap-frozen and liquid nitrogen-stored tumor tissue of five CT-pNEN patients was formalin-fixed and paraffin-embedded (FFPE) and used for IHC. Two-micrometer tumor sections were incubated with commercial mouse monoclonal antibody against *MUC4* (CellMarque™). Staining of *MUC4* epitopes was performed by a manual procedure using the DAKO FLEX EnVision Kit (Agilent, Santa Clara, CA, USA) according to the manufacturer’s instructions. Heat-induced epitope retrieval at pH 9 and 95°C for 30 min was established using an automated decloaking chamber NxGen (Biocare Medical, PAcheco, CA). *MUC4* primary antibody (clone 8G7, BSB2555, diluted 1:200, Bio SB, Santa Barbara, CA, USA) applied for 30 min and visualized by DAKO EnVision™ FLEX DAB+ Substrate Chromogen System (Agilent, Santa Clara, CA, USA) resulting in a brown intracellular signal. *MUC16* (*CA125*, Dako Omnis) was a ready-to-use IVD-labeled mouse monoclonal antibody by an automatic procedure using the Agilent DAKO Omnis instrument (Agilent, Santa Clara, CA, USA) according to the manufacturer’s instructions. Briefly, heat-induced epitope retrieval was at pH 9 and 97°C for 20 min. *MUC16* primary antibody (clone M11, GA701) was applied for 20 min and visualized by DAKO EnVision™ FLEX DAB+ EnV FLEX Substrate Working Solution (Agilent, Santa Clara, CA, USA), resulting in a brown intracellular signal. Stained slides were scanned with the PANNORAMIC slide scanner (3DHISTECH, Budapest, Hungary). Quantitative analysis of IHC was annotated by two genitourinary pathologists.

In this study, 2-µm FFPE tissue slices were stained with primary antibodies against human calcitonin 1:1,000 (A0576, Dako Agilent, Santa Clara, CA, USA), human synaptophysin 1:600 (M7315, Dako Agilent), human chromogranin A 1:2,000 (503-1524 ZYTOMED SYSTEMS, Berlin, Germany), human *Ki-67* 1:200 (M7240, Dako Agilent), human insulin 1:200 (Mob234 Diagnostic BioSystems, Pleasanton, CA, USA), human adrenocorticotropin (ACTH) 1:1,000 (M3501, Dako Agilent), and human insulinoma-associated 1 (*INSM1*) 1:100 (BSB-123 Bio SB, Santa Barbara, CA, USA). The slices were processed by an automated routine device Leica Bond Max (SN 49621/996021 Leica, Fulda, Germany) for standard deparaffinization and staining with primary and secondary antibodies.

**Figure 1:**
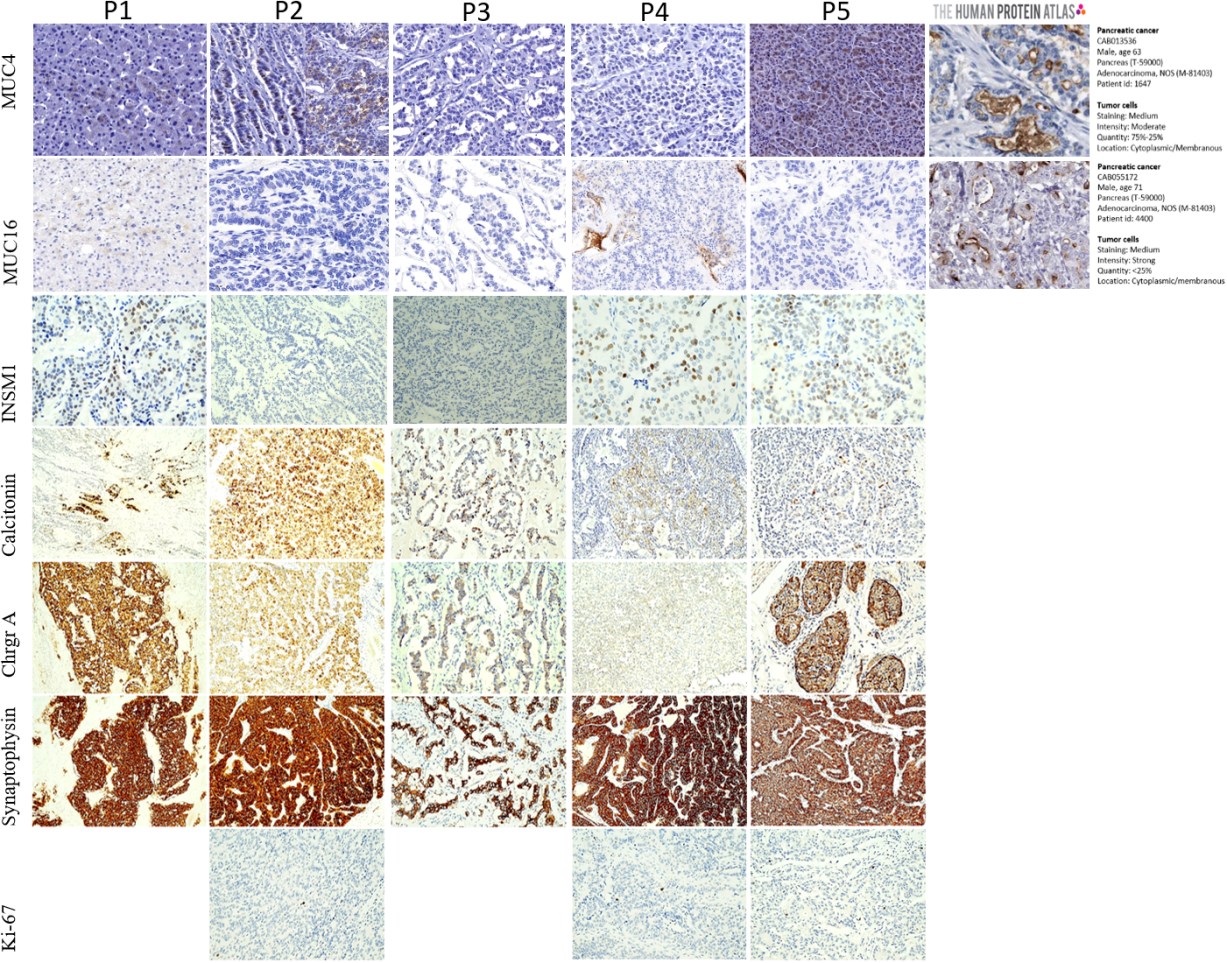
Representative images of immunohistochemistry stainings (IHC) of the calcitonin- producing pancreatic neuroendocrine neoplasms tissue (CT-pNENs), isolated from 5 patients (P1–P5), for Mucin 4 (*MUC4*), Mucin 16 (*MUC16*), human Insulinoma associated 1 (*INSM1*), Calcitonin, Chromogranin A (*Chrgr A*), Synaptophysin and proliferation Marker Protein (*Ki-67*). The staining was performed in primary resected tumor tissue. Magnification 100x (except *MUC4* and *MUC16*: magnification 40x). Patient P1 shows the double positive liver metastasis for staining *MUC4* and *MUC16*. Patient P2 shows *MUC4* cytoplasmic and nuclear staining in the same case. Patient P4 is positive for *MUC16* and Patient P5 for *MUC4*. Patient P3 and P4 are negative for *MUC4* and patient P2, P3 and P5 are negative for *MUC16*. Patient P2 and P3 show a negative staining for *INSM1*.

An experienced pathologist has performed the quantitative analysis (**Figure 1**). The quantification was estimated by sampling the number of positive cells every 1,000 counted cells per tissue area. The total amount of positive cells was calculated in percentage: positive cells/all cells × 100. The presence of adenocarcinoma has been excluded morphologically by an experienced pathologist and by the positive staining of synaptophysin of all samples included in the study.

The HPA database (https://www.proteinatlas.org/, (27)) is a free online database that provides abundant transcriptome and proteome data on human normal or pathological tissues through RNA-sequencing and immunohistochemical analysis. In the present study, the protein expression and distribution of *MUC4* (https://www.proteinatlas.org/ENSG00000145113-MUC4/pathology/pancreatic+cancer#img) and *MUC16* (https://www.proteinatlas.org/ENSG00000181143MUC16/pathology/pancreatic+cancer#img) were investigated in pancreatic cancer tissues and compared with normal tissues in HPA.

## 3 Results

### 3.1 Patients

From January 2009 to December 2019, 140 patients with pNENs underwent surgery at Philipps-University Marburg. These included 76 (54%) nonfunctioning pNENs and 64 (46%) functioning pNENs (30 insulinomas, 24 gastrinomas, 10 rare pNENs), which were sporadic in 94 (67%) cases and associated with *MEN1* (Multiple Endocrine Neoplasia 1) in 46 (33%) cases. These included five (3.6%) patients with CT-pNEN, all of whom were most likely sporadic. Three of the five patients were women; the median age was 64 years (range 54–69).

Two patients presented with upper abdominal pain; one of these patients also suffered from diarrhea. Two other patients were diagnosed with hypercalcitoninemia during a thyroid examination. Further evaluation of a possible medullary thyroid carcinoma by computed tomography revealed then a pancreatic mass, although no abdominal symptoms were present. The fifth patient underwent thyroidectomy at an external hospital for suspected medullary thyroid carcinoma. Since the CT values did not decrease postoperatively, further examination was applied to visualize the pNEN.

Serum CT was elevated in all five patients with a range from 15- to 200-fold above baseline (**Table 1**). Serum chromogranin A levels were also elevated in three patients. Furthermore, all five patients were characterized by positive IHC staining for calcitonin, synaptophysin, chromogranin A. Three out of five samples were positive for *INSM1* too (**Figure 1**). All of the patients were negative for adrenocorticotropin in the histological staining. The possibility of a MiNEN (mixed NEN) has been excluded by the absence of tumor tissue of adenocarcinoma origin.

**Table 1:** Patient characteristics, histology, and follow-up.

Pat.	Gender	Age at Dx (years)	Tumor size (cm)	Preoperative serum CT level (Ref.)	TNM/ tumor cells positive for CT	Postoperative serum CT level (Ref.)	Follow-up (mo.)/ Status
1(471)	F	68	4	103 pg/mL (<5)	pT2 pN0 (0/12), pM1 (Hepar), G2, L1 V1 R2 Hepar); 5%	86.9 pg/mL (<5)	36 DOD
2(649)	M	54	13	1,586 pg/mL (<11.6)	pT3, pN1 (7/22), M0, G2, L0, V1, Pn0, R0; 20%	2.3 pg/mL (<5)	80 NED
3(728)	M	69	7.5	139 pg/mL (<8.4)	pT2, pN0 (0/16), M0, G2, R0, L0, V0, Pn0, R0; 10%	<2 pg/mL (<8.4)	63 NED
4(882)	F	62	11.3	149.5 pg/mL (<9.8)	pT3 pN1 (1/12) pM1 (Hepar), G1, L1 V1 Pn0 R0; 5%	<0.5 pg/mL (<9.8)	23 NED
5(879)	F	67	3.5	88 pg/mL (<9.8)	pT2 pN0(0/16), M0, G2, L0, V0 Pn0 R0; 5%	<0.5 pg/mL (<9.8)	20 NED

Dx, diagnosis; CT, calcitonin; Ref., reference value; mo., months; NED, no evidence of disease; DOD, dead of disease.

Preoperative cross-sectional imaging with computer tomography and MRI demonstrated solitary neoplasms in all patients: three in the pancreatic tail, one each in the body and head. The tumor size ranged from 3.5 cm to 13 cm in diameter. Functional somatostatin-receptor imaging with Ga68-DOTATOC-PET-computertomography visualized all five pNENs. One patient demonstrated lymph node metastasis, two patients had both lymph node and liver metastases, while two patients had no signs of metastasis.

Potentially curative resection was performed in four patients, including distal splenopancreatectomy in three patients and total pancreatectomy in one patient. One of these patients also underwent wedge resection of liver metastasis in combination with radiofrequency ablation, thus resulting in a successful complete resection (R0 situation). The fifth patient with diffuse liver metastasis underwent palliative distal pancreatic resection and postoperative transarterial chemoembolization (TACE) of the liver metastasis and chemotherapy with streptozotocin.

Histopathology examination revealed one G1 and four G2 tumors. Immunoreactivity for CT ranged from 2% to 20% in tumor cells. The surgical and pathologic features are summarized in **Table 1**.

Patients were followed up between 20 and 80 (median 43) months after surgery. At the time of evaluation, four of five patients were alive and had no evidence of disease. One patient (No. 4) had a single lymph node recurrence resected 20 months after initial surgery for stage IV CT-pNEN. The patient with diffuse liver metastasis at the time of palliative surgery died 36 months after surgery due to extensive liver metastasis.

### 3.2 WES data

The sequencing quality of the WES was analyzed, and the raw data, Q30 (proportion of mapped reads), and mean depth of each sample are shown. All samples had Q30 ratio >90%, good sequencing quality, >99% of mapped reads, and an average sequencing depth >200×, which was sufficient to identify mutations. The distribution of these SNVs and INDELs (Insertions/Deletions) and their feature annotation are shown in **Supplementary Tables S1, S2**.

### 3.3 Mutational signatures of CT-pNENs

Since there is no WES database for CT-pNEN tumors available, we first evaluated mutated genes in pNENs. Scarpa et al. (29) performed whole-genome sequencing of 102 sporadic primary pNENs and defined the genomic events that characterize their pathogenesis. We compared their most relevant mutated genes with our WES data from CT-pNEN tumors. Two cases had a somatic *MEN1* missense mutation and one case an *ATRX* (Alpha Thalassemia/intellectual disability syndrome X-linked) missense mutation. Mutations of other SNVs were found both in the tumor and the corresponding control DNA derived from the patient serum (**Table 2**). Only one case demonstrated a *ULK1* mutation with clinical significance [SIFT (D), PolyPhen (D), FATHMM (T)]. The other mutations had no effect on the protein-coding function or were unknown for clinical relevance. The germline *BRCA2* mutations were additionally compared with the BRCA2 database (https://arup.utah.edu/database/​BRCA/Home/BRCA2_landing.php) for clinical relevance.

**Table 2:** Germline and missense single nucleotide variations (SNV) in genes mutated in calcitonin- producing pancreatic neuroendocrine neoplasms tissue (CT-pNEN) tumors previously described in other pancreatic neuroendocrine neoplasms tissue (pNEN) (28-33).

Gene	Mutation type	Position	ClinVar	Patient ID
**ATRX**		chrX:77682471	Benign	T879, T882, T728
**BRCA2**	c.1114A>C	chr13:32332592	Benign	T879, T882, T728, T471, T649
**BRCA2**	c.7397C>T	chr13:32355250	Benign	T879, T882, T728, T471, T649
**CDKN2A**	c.442G>A	chr9:21970917	Benign	T879
**MEN1**		chr11:64804546	Benign	T879, T882, T728, T471, T649
**MEN1**		chr11:64808033		T471
**PTEN**		chr10:87864144		T879, T471
**SETD2**		chr3:47083895	not_provided	T879, T882, T728, T471, T649
**SETD2**		chr3:47121396	not_provided	T649
**TP53**	c.215G>C	chr17:7676154	drug_response	T879, T882, T471, T649
**TSC1**	c.965T>C	chr9:132911517	Benign	T879
**ULK1**		chr12:131917021		T649, T728
**ULK1**		chr12:131917512		T649
**ULK1**		chr12:131918616		T879, T882, T728, T471, T649
**MUTYH**		chr1:45334484		T471

Furthermore, no clinically relevant DNA- or protein-damaging mutations were found in the genes CALCA (calcitonin) and CALCR (calcitonin receptor). Interestingly, neither gene was responsible for causing the aberrant expression of *CALCA* in the CT-pNEN tumors.

#### 3.3.1 Pathogenic variations in CT-pNEN tumors

After filtering the SNVs with ClinVar “pathogenic” classification from CT-pNEN samples, our next focus was on commonly known pathogenic variations. The mTOR pathway-related gene *PIK3CA* (phosphatidylinositol-4,5-bisphosphate 3-kinase, catalytic subunit alpha) was the only somatic pathogenic SNV found in one patient. All other pathogenic SNVs were also found in control DNA. Seventeen genes, including *FGFR4* (Fibroblast Growth Factor Receptor 4), *TYMP* (Thymidine Phosphorylase), *ABCA4* (ATP Binding Cassette Subfamily A Member 4), *COQ4* (Coenzyme Q4), *DPYD* (Dihydropyrimidine Dehydrogenase), *KLKB1* (Kallikrein B1), and *TGFBI* (Transforming Growth Factor Beta Induced), were characterized by 20 mutations (**Table 3**). Notably, the *TYMP* (c.866A>C), *ABCA4* (c.5338C>G), COQ4 (c.718C>T), and *TGFBI* (c.1501C>A) were predicted as “D (deleterious)” by SIFT, Polyphen2, and FATHMM software. Three genes were recurrently mutated in three CT-pNENs patients. *DPYD* (c.85T>C), *FGFR4* (c.1162G>A) and *KLKB1* (c.428A>G) mutations classified as pathogenic, but SIFT, Polyphen2, and FATHMM all do not predict *KLKB1* as “D (deleterious).” None of the encoded exons had pathogenic INDELs. The next focus was on the germline and pathogenic SNVs that either occur in at least three patients or were predicted as “D (deleterious).”

**Table 3:** Pathogenic, germline and missense single nucleotide variations (SNV) in genes mutated in calcitonin- producing pancreatic neuroendocrine neoplasms tissue (CT-pNEN) tumors not previously described in other pancreatic neuroendocrine neoplasms tissue (pNEN) (28-33).

Gene	Mutation type	Position	ClinVar	Patient ID
**ABCC6**	c.3803G>A	chr16:16157742	Pathogenic	T882
**ABCC6**		chr16:16185006	Pathogenic	T882, T728
**ABCC6**		chr16:16187150	Pathogenic	T882
**CST3**	c.73G>A	chr20:23637790	Pathogenic	T879, T882
**HFE**	c.187C>G	chr6:26090951	Pathogenic,_other,_risk_factor	T882
**IL4R**	c.223A>G	chr16:27344882	Pathogenic,_protective	T649, T879
**CCDC114**	c.742G>A	chr19:48303953	Pathogenic	T879
**TYMP**	c.866A>C	chr22:50526638	Pathogenic	T879
**STOX1**	c.1824A>C	chr10:68885620	Pathogenic	T879
**ABCA4**	c.5338C>G	chr1:94014665	Pathogenic	T728
**PIK3CA**		chr14:94378610	Pathogenic/Likely_pathogenic	T471
**DPYD**	c.85T>C	chr1:97883329	Pathogenic	T728, T879, T882
**APOA4**		chr11:116820918	Pathogenic	T649
**C1GALT1C1**		chrX:120626774	Pathogenic	T879, T882
**COQ4**	c.718C>T	chr9:128333565	Pathogenic	T882
**TGFBI**	c.1501C>A	chr5:136055770	Pathogenic	T649
**ASB10**		chr7:151186916	Pathogenic	T879
**FGFR4**	c.1162G>A	chr5:177093242	Pathogenic	T471, T728, T879
**SERPINA1**		chr3:179218294	Pathogenic	T471
**KLKB1**	c.428A>G	chr4:186236880	Pathogenic	T649, T728, T879

The ClinVar filter “pathogenic” is very stringent, and the ClinVar classification is for most of the mutations unknown. In a second approach, the somatic SNVs were filtered with SIFT, Polyphen2, and FATHMM, which predicted them as “D (deleterious).” Except for *ATRX* and *MEN1*, which are known to be commonly mutated in pNENs (28–32), we identified novel mutation candidates in this patient cohort. *MEN1* mutation is the only one found in two patients, which is commonly mutated in pNEN (**Table 4**). All variants (germline variants highlighted in yellow) found in the individual tumor cases in annotated genes can be found in **Supplementary Table S3**.

**Table 4:** Potentially deleterious, somatic and missense single nucleotide variations (SNV) of calcitonin- producing pancreatic neuroendocrine neoplasms tissue (CT-pNEN) tumors.

Gene	Position	ExAC_ALL	ClinVar	Patient ID
**ABCC11**	chr16:48205534	0		T471
**ATP4A**	chr19:35560401			T882
**ATRX**	chrX:77557543			T882
**CAV3**	chr3:8745665		Uncertain_significance	T882
**CCBL2,RBMXL1**	chr1:88983615	0.3593		T649
**CHKA**	chr11:68120969			T471
**CNN2**	chr19:1037719	0.0003		T649
**COL5A3**	chr19:9970672			T471
**FARP1**	chr13:98390051			T882
**GP9**	chr3:129062079			T882
**GRHPR**	chr9:37424916	0		T471
**HES4**	chr1:999786			T649
**HSPG2**	chr1:21862027			T882
**MEN1**	chr11:64806322			T649, T471
**SLC35G5**	chr8:11332020	0.0079		T471

The somatic single nucleotide variations (SNVs) were filtered by with SIFT, Polyphen2 and FATHMM, whichpredicted them as “D (deleterious)”.

Overall, the HRD score of all tumor cases was determined, where all tumor cases have a low HRD score between 2 and 30.

#### 3.3.2 Mutations across mucins

We found several mutated mucins in CT-pNEN tumors. Mucins are large multifunctional glycoproteins whose primary functions are to protect and lubricate the surfaces of epithelial tissues lining ducts and lumens within the human body. Several lines of evidence also support the involvement of mucins in more complex biological processes such as epithelial cell renewal and differentiation, cell signaling, and cell adhesion. Recent studies have uncovered the role of selected mucins in the pathogenesis of cancer. Deregulated mucin production has been associated with numerous types of cancers and inflammatory disorders (33). However, mucins are specific markers for adenocarcinoma and not for neuroendocrine neoplasms. Here, the mutation pattern was heterogeneous across the five tumor samples (**Supplementary Figure S1**). For *MUC4*, we found mutations in all five cases and for *MUC16* in four cases. Both membrane-bound mucins are large genes with 27 exons (*MUC4*) and 86 exons (*MUC16*), respectively. The SNV mutation patterns are shown in **Supplementary Figure S2A, S2B**. The clinical significance (ClinVar) of *MUC4* and *MUC16* SNVs is unknown. To address the question whether *MUC4* and *MUC16* SNVs have an impact on protein expression, immunohistochemistry of both genes was performed. *MUC16* (known as CA125), a well-established antibody, is a useful tool for classification of a variety of tumors, such as adenocarcinomas of the colon, breast carcinomas, malignant mesothelioma, uterine adenomatoid tumor, lung bronchoalveolar carcinoma, ovarian endometrioid and serous carcinomas. *MUC4* is generally not detectablein normal pancreas, but is highly expressed in the vast majority of pancreatic neoplasms, such as pancreatic ductal adenocarcinoma (34). The liver metastasis in one patient was double-positive for *MUC4* and *MUC16*, while two patients stained only positive for *MUC4*, and another patient was only positive for *MUC16* (**Figure 1**). The immunohistochemistry stainings of *MUC4* and *MUC16* expression in all five CT-pNENs cases, including positive and negative staining controls, are shown in **Supplementary Figure S3**.

### 3.4 DAVID functional gene enrichment analysis

For the functional gene enrichment analysis, we used germline SNVs (average 76 SNVs per case) filtered with Polyphen2=D. Four of the five analyzed cases (80%) show an overrepresentation of the *KRT* gene family. *KRT3*, *KRT18*, and *KRT32* were found in four cases (80%). *KRT8*, *KRT35*, *KRT75*, *KRT77*, and *KRT81* were found in three cases (60%).

## 4 Discussion

CT-pNENs are considered extremely rare, where the 3.6% (n = 5) prevalence in our patient cohort most likely reflects the referral bias of an European Neuroendocrine Tumor Society (ENETS) center of excellence. This present study demonstrated, for the first time, that a small cohort of patients (n = 5) affected by CT-pNENs has a unique molecular genetic signature compared to other pNEN subtypes, particularly affecting *FGFR4*, *DPYD*, *MUC4*, *MUC16*, and *KRT* family genes (28, 35). Elevated serum CT levels are usually indicative for Medullary Thyroid Carcinoma (MTC), where only in very rare occasions it is caused by CT-pNENs (8). The 1990 analysis by Eriksson et al. (35) highlighted that CT-pNENs cause only a modest increase in CT serum levels, approximately twice the upper limit of the reference value. In the present study, however, the CT elevation was at least 15-fold of the upper reference in all five patients.

The high risk for malignancy of CT-pNENs was also seen in the present study, where metastatic disease was pathologically confirmed in three of five patients. However, an aggressive surgical approach leads to survival of four out of five patients with no evidence of disease after a mean follow-up of 45 months. Thus, extrapolation of these data will suggest a 5-year survival rate comparable to that reported for vipomas, pancreatic gastrinomas, and nonfunctional pNENs larger than 2 cm in size (36, 37).

In contrast to insulinomas and nonfunctioning pNENs, the molecular profile of CT-pNENs has not been explored yet (28–31). WES generally has high sensitivity for common, rare, and low-frequency mutations. It can find most disease-related mutations in the exon region and only requires to sequence approximately 1% of the genome (38). WES had been previously used to detect the mutational landscape of pNENs, mostly the nonfunctioning subtype, and provided novel insights into the pathogenesis of the disease (28–31). Similar to other studies, we detected somatic *MEN1* and *ATRX* mutations in two and one patient of CT-pNENs, respectively. *MEN1* and *ATRX* are the most common mutations of other pNENs (37% and 10%) (28–31). The mTOR pathway gene *PIK3CA* showed a somatic mutation in one CT-pNEN patient and was rarely mutated in other pNENs (1.4%) (29, 39).

The current results showed that CT-pNENs have a unique molecular signature compared to other pNENs, particularly within a distinct mutational landscape of *MUC* family genes. This is consistent with previous studies that have suggested up to four distinct molecular subtypes of pNENs (40, 41). Interestingly, the expression of *MUC*s is normally considered a specific marker for adenocarcinoma. Here, for the first time, it has been highlighted that the genetic alterations occurring at those genes caused the expression of *MUC4* (three cases) and *MUC16* (two cases) proteins in five CT-pNENs. The mutations occurring at the *MUC4* and *MUC16* genes were partially able to determine the reexpression of an immunohistochemically detectable protein product, thus highlighting that the mutation pattern represents a valid tool to build up the genetic profile of the patient. Nevertheless, the effect of the mutations, including the protein product, and their activity and stability should be included in the patient profile.

Of note, the immunohistochemical overexpression of calcitonin, detected by a specific antibody solely binding to calcitonin thus excluding any possible binding with the calcitonin gene-related peptide (CGRP), could not be explained by the mutations occurring at the *CALCA* or *CALCR* genes. Instead, it could be related to possible epigenetic modifications that have not been analyzed in this study. A further analysis, conducted by following the WHO (World Health Organization) standard classification (42), confirmed the pancreatic neuroendocrine origin of the neoplastic tissue resected from the patients. In particular, all of the samples were positively stained for synaptophysin, chromogranin A, and part of the samples positive for *INSM1* (nuclear, three of five samples) and were negatively stained for insulin and adrenocorticotropin. Furthermore, the absence of adenocarcinoma cells excluded the possible presence of MiNEN (mixed NEN).

Here, we identified novel potentially deleterious somatic SNVs in genes such as *ABCC11*, *ATP4A*, *CAV3*, *CHKA*, *CNN2*, *FARP1*, *GP9*, *GPHPR*, *HES4*, and *SLC35G5*. *ABCC11* (ATP Binding Cassette Subfamily C Member 11) associated with diseases including apocrine gland secretion and lateral sinus thrombosis. Among its related pathways are transport of glucose and other sugars, bile salts and organic acids, metal ions and amine compounds, and *CDK*-mediated phosphorylation and removal of *Cdc6* (43). The protein encoded by *ATP4A* belongs to a family of P-type cation-transporting ATPases. Loss-of-function mutations in a subunit of the parietal cell proton pump (*ATP4A*) cause familial gastric NET (44). *CAV3* is a member of caveolin family proteins, which functions as a component of the caveola plasma membranes found in most cell types. Mutations identified in this gene lead to interference with protein oligomerization or intracellular routing, disrupting caveola formation and resulting in Limb-Girdle muscular dystrophy type-1C, hyperCKemia, or rippling muscle disease (45). Diseases associated with *CHKA* (Choline Kinase Alpha) include muscular lipidosis and hepatitis C virus infection (46). *CHKA* was found to be overexpressed in 90% of pancreatic tumors (47). *CNN2* encodes for an alpha chain of one of the low-abundance fibrillar collagens. Mutations in this gene are thought to be responsible for the symptoms of a subset of patients with Ehlers–Danlos syndrome type III (48). *FARP1* (pleckstrin domain protein 1) expression is related to poor prognosis of advanced gastric cancers (49).

Genomic alterations and mutation patterns in mucins show an important role for the development of novel biomarkers and therapeutic agents against cancers (50). King et al. reviewed the The Cancer Genome Atlas (TCGA) database and extracted mucin mutation patterns across 11 mucin-expressing tissue, and they showed each tissue has its own mucin signature. In pancreatic adenocarcinoma, the mucin signature has an impact on overall survival (51), and there is a potential use of the mucin expression pattern in the diagnosis of pancreatic neoplasm (52). The mutation pattern in mucins of CT-pNEN tumors was completely unknown. There are 22 mucin genes encoding large O-glycoproteins divided into two major subgroups: membrane-bound and secreted mucins. In previous genomic analyses, only very few cases of pNEN (2%–4%) showed *MUC4* and *MUC16* mutations (29).

Several potentially deleterious germline SNVs that had not been previously described were also discovered in the CT-pNENs. *TYMP*, mutated in three of five CT-pNENs, encodes an angiogenic factor that promotes angiogenesis *in vivo* and stimulates the *in vitro* growth of a variety of endothelial cells. Mutations in this gene have been associated with mitochondrial neurogastrointestinal encephalomyopathy (53). The protein of *COQ4*, mutated in one of five CT-pNENs, encodes a component of the coenzyme Q biosynthesis pathway. Coenzyme Q shuttles electrons between complex I or II and complex III of the mitochondrial transport chain. Mutations in this gene are associated with mitochondrial disorders linked to coenzyme Q deficiency (54). The protein of *TGFB1*, mutated in one of five CT-pNENs, plays a role in cell–collagen interactions and is induced by transforming growth factor-beta. Mutations in this gene are associated with multiple types of corneal dystrophy (55). This gene encodes a glycoprotein that participates in the surface-dependent activation of blood coagulation, fibrinolysis, kinin generation, and inflammation. Certain mutations in *KLKB1*, mutated in one of five CT-pNENs, cause prekallikrein deficiency (56), and *KLKB1* was mutually exclusive with *ATRX*, *DAXX*, and *MEN1* of pancreatic neuroendocrine tumors (PanNets) (57, 58).

Because we only have WES data for five cases, causal interpretation of the mutational landscape in CT-pNEN is difficult. Further research is needed to explain differences in pathogenesis compared with CT-pNENs. In particular, multi-omics data such as RNA-sequencing, methylation, and whole-genome sequencing could be informative.

## Data availability statement

The datasets presented in this study can be found in online repositories. The names of the repository/repositories and accession number(s) can be found below: https://www.ncbi.nlm.nih.gov/gap/, phs003060.v1.p1.

## Ethics statement

The studies involving humans were approved by the ethics committee of the University of Marburg (No:206/10 and No:104/99). The studies were conducted in accordance with the local legislation and institutional requirements. The participants provided their written informed consent to participate in this study. Written informed consent was obtained from the individual(s) for the publication of any potentially identifiable images or data included in this article.

## Author contributions

DB designed and performed the research, revised and analyzed the clinical data, and wrote the article. PW performed the research, characterized the histological samples, and assisted in the correction of the paper. CD designed and performed the research, performed the bioinformatics and statistical analysis, and wrote the article. AR provided advice and collected and characterized the histological samples. KB provided advice, scanned the histological samples, and assisted in the correction of the paper. KP collected and analyzed the clinical patient data. PD performed the DNA isolation, clinical data collection, arranging of DNA sample processing, and article revision. CB collected the tissue samples, performed the DNA isolation, and analyzed the histological samples. All authors contributed to the article and approved the submitted version.
