# Supplementary material for: Whole-exome sequencing of calcitonin-producing pancreatic neuroendocrine neoplasms indicates a unique molecular signature
Source: Front Oncol. 2023 Sep 12;13:1160921. doi: 10.3389/fonc.2023.1160921 (PMC10522832; doi:10.3389/fonc.2023.1160921)
Supplement: Supplementary file 1 [file DataSheet_1.docx]

**Supplementary material**

Table 1: Single nucleotide polymorphism (SNP) Function and Features statistic of all samples.


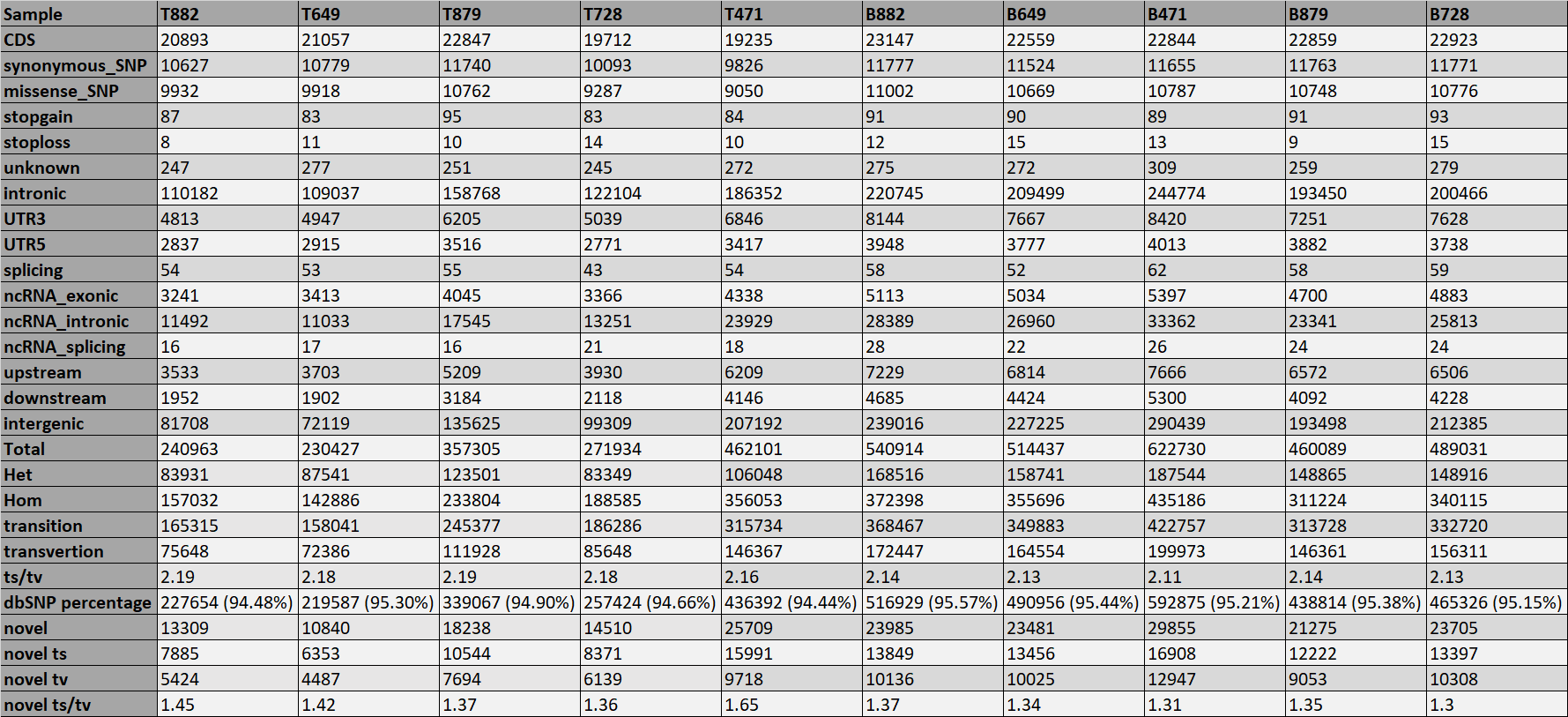


Table 2: Insertions/deletions (INDEL) Function and Features statistic of all samples.


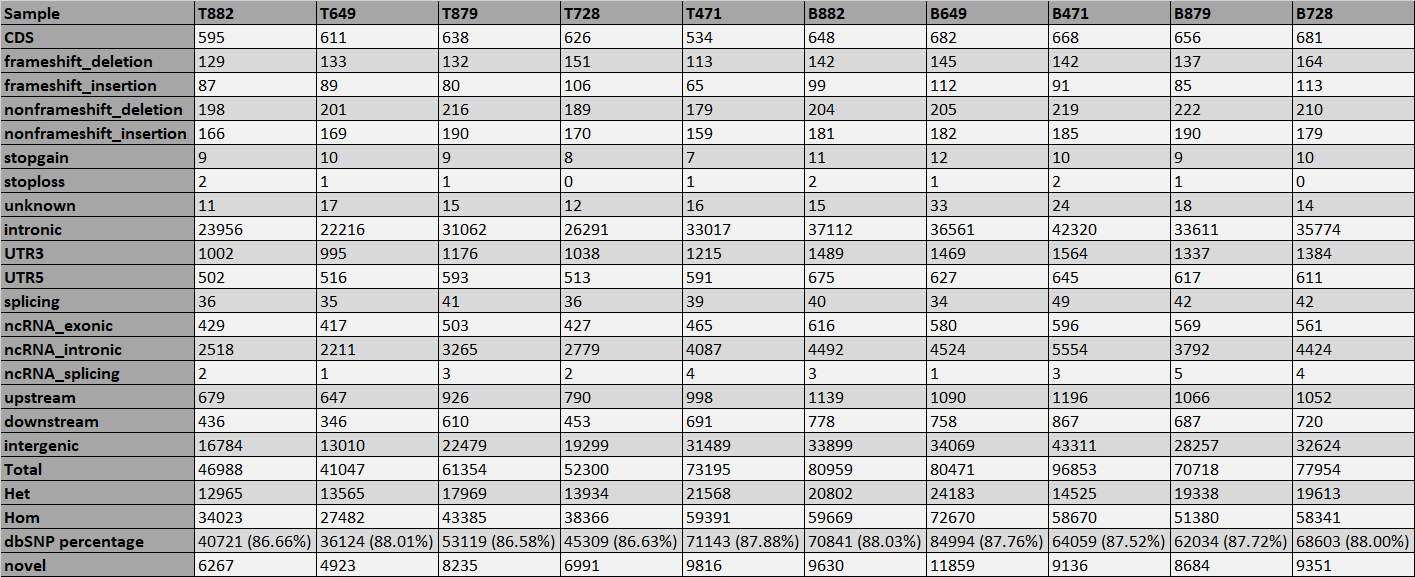


Figure 1: CircosPlot of all *MUC* gene family members where missense SNVs were found (R v. 4.0.5 [59], package RCircos v. 1.2.2). The SNVs are annotated by the following short hand notation: *MUC**_count(germline)_count(somatic).


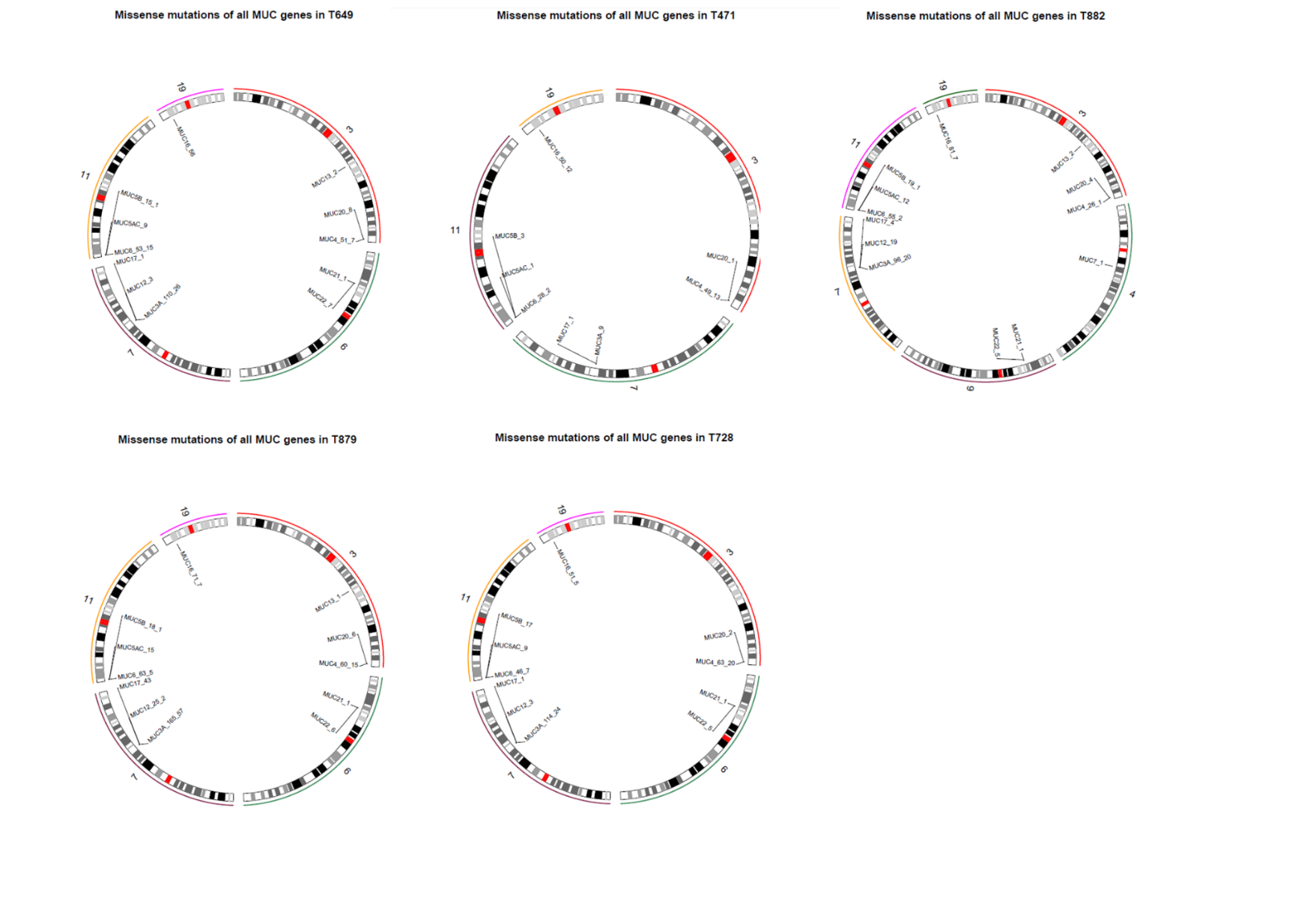


Figure 2A: Lolliplot of all *MUC4* missense and Polyphen2 = D SNVs (R v. 4.0.5 [59], package trackViewer v. 1.26.2). SNVs highlighted in red were somatic SNVs.

*
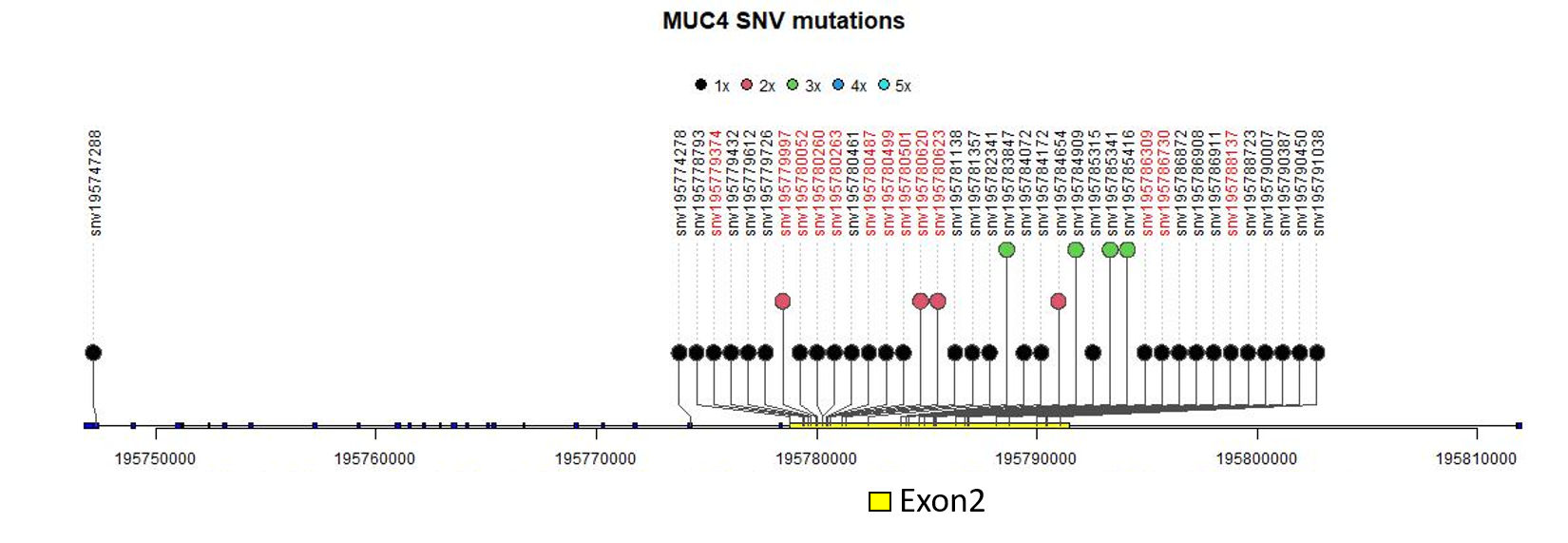
*

Figure 2B: Lolliplots of all *MUC16* missense and Polyphen2 = D SNVs (R version 4.0.5 [59], trackViewer_1.26.2). SNVs highlighted in red were somatic SNVs.


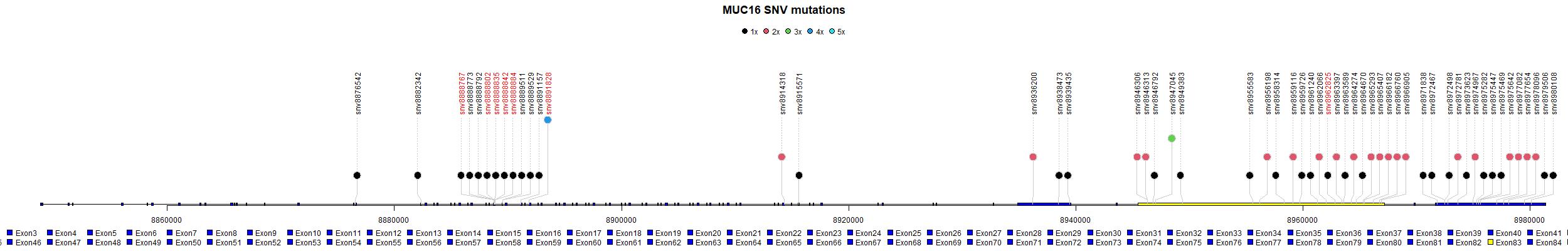


Figure 3: Immunohistochemistry staining’s of *MUC4* and *MUC16* expression of all five CT-pNENs cases (P1-P5) with positive (*MUC4* positive control: colon tissue, *MUC16* positive control: AEG tumor) and negative (liver tissue) staining controls (magnification 40x).

**
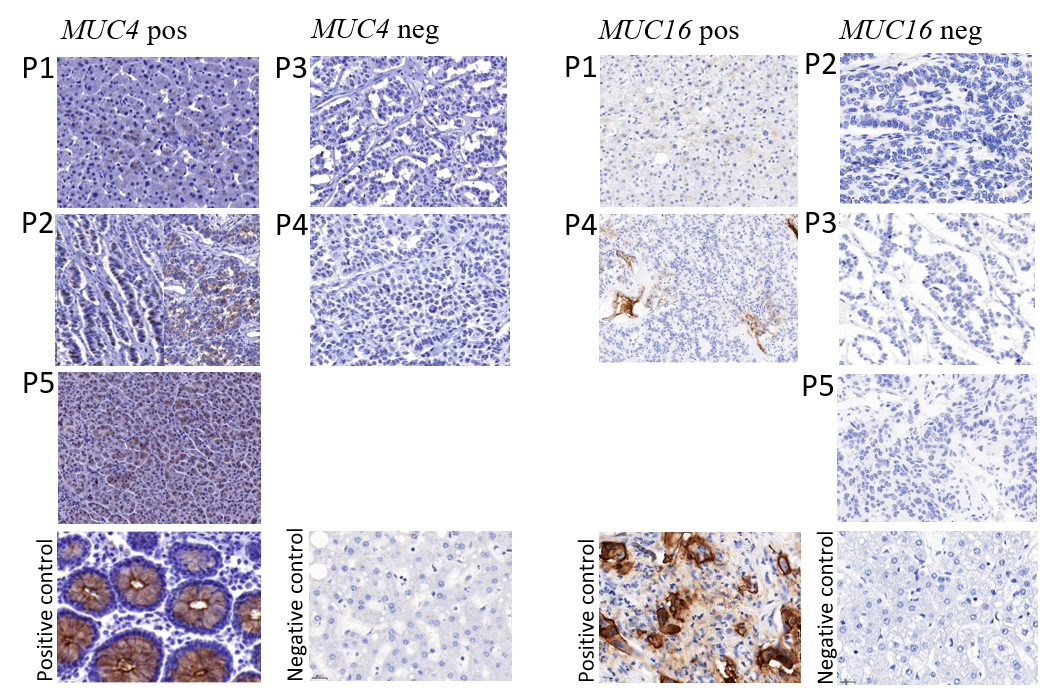
**
